# Association between health literacy and glycemic control in elderly patients with type 2 diabetes and modifying effect of social support

**DOI:** 10.31744/einstein_journal/2020AO5572

**Published:** 2020-11-25

**Authors:** Jonas Gordilho Souza, José Marcelo Farfel, Omar Jaluul, Márcia Silva Queiroz, Márcia Nery

**Affiliations:** 1 Universidade Federal da Bahia Faculdade de Medicina da Bahia SalvadorBA Brazil Faculdade de Medicina da Bahia, Universidade Federal da Bahia, Salvador, BA, Brazil.; 2 Universidade de São Paulo Faculdade de Medicina São PauloSP Brazil Faculdade de Medicina, Universidade de São Paulo, São Paulo, SP, Brazil.; 3 Universidade de São Paulo Faculdade de Medicina Hospital das Clínicas São PauloSP Brazil Hospital das Clínicas, Faculdade de Medicina, Universidade de São Paulo, São Paulo, SP, Brazil.

**Keywords:** Health literacy, *Diabetes mellitus* , type 2, Blood glucose, Aged, Health of the elderly

## Abstract

**Objective:**

To investigate the association between inadequate functional health literacy, considering the *Short Assessment of Health Literacy for Portuguese-speaking* Adults, and glycemic control in elderly patients with type 2 diabetes, and to examine this association in low social support settings, according to *Medical Outcomes Study* .

**Methods:**

Cross-sectional study conducted at the diabetes referral center of a university hospital. Participants were recruited among type 2 diabetes patients aged 60 years or older, between May 2013 and November 2014. The primary outcome was the most recent glycated hemoglobin value measured within the last 6 months.

**Results:**

A total of 398 elderly patients with type 2 diabetes were evaluated. Of these, 232 were not eligible to participate. The final sample comprised 166 participants. Hierarchical multiple linear regression was performed. The following variables were entered in three blocks: sociodemographic characteristics, clinical variables and health literacy scores. Regression analysis of the interaction between health literacy and social support as a determinant of glycemic control was also performed. Mean age of subjects was 68.0 years (standard deviation of 5.9). Mean glycated hemoglobin value was 8.5% (standard deviation of 1.4). Short assessment of health literacy for Portuguese speaking adults score was independently associated with glycated hemoglobin (B=-0.059; p=0.043). The interaction between social support and health literacy score (p=0.003) was a determinant of glycemic control.

**Conclusion:**

Health literacy is associated with glycemic control. Social support may modify the relation between health literacy and glycemic control.

## INTRODUCTION

Health literacy is defined as the degree to which individuals have the capacity to obtain, process, and understand basic health information and services required to make appropriate health decisions. The current concept includes communication and critical skills, such as understanding of medication dosage, labels and exams, search of health information, risk and benefit analysis, communication of needs and discussion of preferences.^(^^1^^)^

Lower rates of health literacy have been observed in some population groups, especially those comprising individuals with low socioeconomic status and older adults. Health literacy may also have a significant impact on elderly individuals with multimorbidities, who require complex therapies and are more likely to use health services.^(^^2^^)^

Several health literacy assessment instruments have been developed in the last decades, including the Short Assessment of Health Literacy for Portuguese-speaking Adults (SAHLPA). This test assesses functional health literacy and is thought to be more appropriate for application in developing countries, since it addresses more rudimentary health literacy-related skills and may thus be more easily administered to individuals with low levels of education.^(^^3^^,^^4^^)^

Associations between inadequate health literacy and conditions, such as poor ability to understand and follow medical instructions,^(^^5^^)^ higher risk of hospital admission^(^^6^^)^ and higher mortality rates^(^^7^^)^ have been demonstrated.

These findings make investigation of associations between health literacy and chronic disease control an important field of research.^(^^8^^)^ Type 2 *diabetes mellitus* (DM2) is thought to be a good model for study of such relations, since it is a complex disease that requires self-care and proper understanding of pharmacological and non-pharmacological treatments, particularly among individuals with longstanding disease. Also, major barriers, such as polypharmacy and pharmacodynamic and pharmacokinetic changes, can make glycemic control difficult in elderly individuals.^(^^9^^)^

Potential relations between health literacy and glycemic control have been investigated by several researchers, with different results.^(^^10^^)^ A recent meta-analysis revealed associations between health literacy and glycemic control in subgroups with DM2 and a higher proportion of subjects with low levels of education.^(^^11^^)^ Low socioeconomic status stands out in most relevant publications reporting this association.^(^^12^^–^^14^^)^

Associations between low health literacy and glycated hemoglobin (HbA1c) levels ≥8% in elderly diabetic patients (odds ratio – OR=4.78; 95% of confidence interval – 95%CI: 1.38-16.6) are reported. Still, glycemic control was not worse among illiterate participants, suggesting difficulties faced by these individuals are more easily identified by family members and health professionals, which may translate into better social support and counteracting measures. However, that study^(^^12^^)^ failed to demonstrate the impact of social support. Osborn et al., investigated the effects of potential determining factors of glycemic control in elderly individuals with high levels of education, and concluded health literacy had only an indirect effect through social support. This finding reinforces the hypothesis that social support may counteract the effects of associations between health literacy and glycemic control.^(^^15^^)^

Few studies to date have investigated mechanisms through which social support may mask or modify the effect of health illiteracy on diabetes control, particularly among elderly patients living in developing countries where levels of education are low. Given the high complexity of DM2 treatment and the greater vulnerability of elderly individuals with lower levels of education to low health literacy, this study set out to examine associations between functional health literacy and glycemic control in elderly patients with DM2 and low levels of education living in a developing country.

## OBJECTIVE

To evaluate the association between functional health literacy (Short Assessment of Health Literacy for Portuguese-speaking Adults) and glycemic control in elderly diabetics with a low level of schooling and living in a developing country. As a secondary objective, we propose an evaluation of the relationships between social support, functional health literacy, and glycemic control in elderly diabetics.

## METHODS

### Design, settings and ethical considerations

An observational, cross-sectional study conducted in a public outpatient clinic. This study was approved by the local institutional ethics committee ( *Comissão de Ética para Análise de Projetos de Pesquisa* – CAPPesq) of *Hospital das Clínicas* of *Faculdade de Medicina* of *Universidade de São Paulo* (USP), protocol number 10639, CAAE: 15560213.9.0000.0068. All patients signed an informed consent form prior to enrollment.

Medical visits were carried out by resident physicians and supervised by diabetes management specialists. Participants had equal access to antidiabetic medications. Medication costs were covered by the local public health system.

### Participants and data collection

Participants were recruited by convenience sampling among patients visiting outpatient clinic of a large diabetes referral center belonging to a tertiary university hospital ( *Hospital das Clínicas* of *Faculdade de Medicina* of USP), in the city of São Paulo (SP), Brazil. Diabetic patients seen between May 2013 and November 2014 were invited to participate at the time of medical visit.

One of the researchers was in charge of recruitment and data collection. Patients were approached in the waiting room prior to medical visits and duly informed about the study. Once informed consent was obtained, participants were interviewed in consultation rooms. Confidentiality was guaranteed.

### Inclusion and exclusion criteria

Inclusion criteria were as follows: 60 years of age or older, fluency in the Portuguese language, type 2 diabetes diagnosis according to American Diabetes Association (ADA) criteria,^(^^16^^)^ and recent HbA1c measurement (up to 6 months prior to enrollment).

Patients were excluded if medical records/examinations revealed the following:

– Less than three visits to the diabetes outpatient clinic.
– Glycated hemoglobin <6.5%, bearing in mind the u-shaped glycemic control curve in elderly patients where extremes are associated with higher mortality.^(^^16^^)^ This criterion was established in order to maintain a linear relation between HbA1c levels and negative outcomes.
– Vision, hearing or speech impairment severe enough to interfere with questionnaire completion. Visual acuity was measured using the Snellen test;^(^^17^^)^ the cut-off value for vision impairment and exclusion from the study was set at 0.5. Hearing ability was assessed by the whisper test;^(^^18^^)^ hearing loss was defined as perception of words or numbers below 50%.
– Use of medications associated with poorly controlled blood glucose levels; previous bariatric surgery or participation in clinical trials investigating diabetes treatment; frailty syndrome, since a less stringent glycemic control target is proposed for frail elderly relative to non-frail individuals.^(^^16^^)^ Frailty syndrome was defined according to criteria: unintentional weight loss greater than 5% in the last year; inability to sit and rise from a chair five times without support; loss of energy defined by the question: “Do you feel full of energy?”. Individuals satisfying two out of these three criteria were defined as frail.^(^^19^^)^
– Cognitive impairment defined as deficits in the Mini Mental State Examination (MMSE). Mini Mental State Examination scores were combined with level of education. The following cut-off values were applied according to years of formal education:^(^^20^^)^ illiteracy and MMSE score equal to or lower than 21; 1 to 5 years of education and MMSE score of 22 or lower; 6 to 11 years of education and MMSE score of 23 or lower; higher level of education (12 years or more) and MMSE score of 24 or lower.
– Prior diagnosis of confusional state, psychotic disorders, mania, alcoholism or drug use; laboratory abnormalities that may interfere with HbA1c reading method, such as thyroid dysfunction (thyroid stimulating hormone <0.1 or >10mU/L),^(^^21^^)^ anemia (hemoglobin <11mg/dL and <10mg/dL, men and women respectively),^(^^22^^)^ chronic kidney disease with estimated glomerular filtration rate below 30mL/min/1.73m^2^, determined using the Cockcroft-Gault formula^(^^23^^)^ and chronic parenchymal liver disease classified as Child-Pugh B or C.^(^^24^^)^

### Data collection tools and variables

#### Demographics, physical and clinical evaluation

The first pieces of information extracted from medical records and/or interviews were time since diagnosis of diabetes and prescribed drugs in use. Sociodemographic data such as age, sex, marital status (married or not), years of education, race as determined by the interviewer (white or non-white) and previous occupation (manual labor or not) were also collected. Socioeconomic status was determined according to Brazilian Criteria of Economic Classification (BCEC) and expressed as interval scales.^(^^25^^)^

Glycated hemoglobin values obtained over the last 6 months were extracted from medical records. In our service, HbA1c is measured using the high-performance liquid chromatography (HPLC) method certified by the National Glycohemoglobin Standardization Program.

Anthropometric assessment was carried out using a digital scale (Lucastec, Brazil). Body mass index (BMI) was calculated based on height and body weight measurements.

Instruments used in this study are described in table 1 .

**Table 1:** Instruments used for evaluation and interpretation of variables

Instrument	Portuguese version	Range	Interpretation
SAHLPA short version – evaluates health literacy	Apolinario et al.^(^^4^^)^	0-18	Analyzed as an interval variable. Can be analyzed as a dichotomous variable; scores <14 define poor health literacy
SKILLD – evaluates diabetes knowledge	Souza et al.^(^^26^^)^	0-10	Analyzed as an interval variable. No cut-off value
MOS – evaluates 5 social support dimensions	Griep et al.^(^^27^^)^	0-95	Analyzed as an interval variable; data sets divided into terciles. No cut-off value
MRCI – evaluates pharmacological treatment complexity	Melchiors et al.^(^^28^^)^	Up to 0	Analyzed as an interval variable. No cut-off value
GDS-15 – evaluates symptoms of depression	Almeida et al.^(^^30^^)^	0-15	Participants scoring >5 were considered depressed

SAHLPA: Short Assessment of Health Literacy for Portuguese-speaking adults - short version. SKILLD: Spoken Knowledge in Low Literacy Patients with Diabetes; MOS: Medical Outcomes Study; MRCI: Medication Regimen Complexity Index; GDS-15: Geriatric Depression Scale.

#### Health literacy and diabetes knowledge

Health literacy assessment was based on SAHLPA *scores* . This tool analyzes functional health literacy via reading of medical terms that must be correlated with two other words. Upon reading the word “osteoporosis”, for example, the interviewee must choose between two alternatives (“bone” or “muscle”).^(^^3^^,^^4^^)^ Reasons for choosing SAHLPA were proper validation for the Portuguese language and simplicity, which makes it easier to apply to individuals with rudimentary literacy skills. The short version of this test is intended for elderly individuals, given it as accurate as the long version (50 items) but more user friendly in the context of comprehensive geriatric assessment. In this study, SAHLPA scores were analyzed as intervals.

Diabetes knowledge was measured using SKILLD. This instrument consists of ten questions inquiring about understanding of the disease. Given it is an orally administered test, reading ability is of little importance. Hence the suitability for populations with low levels of education. SKILLD scores range from 0 to 100%; the higher the score, the better the understanding of diabetes-related issues.^(^^26^^)^

#### Social support

The need for help with medications was investigated in medical records. Participants were categorized as in need of help to organize, remember or manage their medications or fully dependent.

The Medical Outcomes Study (MOS) instrument was also used. This instrument evaluates five dimensions of social support received by patients, with scores ranging from 1 (never) to 5 (always).^(^^27^^)^ Final scores range from zero to 95, where 95 corresponds to the best possible social support. No cut-off points have been described in literature. Therefore, scores were split into terciles for interaction analysis.

### Medication use and adherence

The Medication Regimen Complexity Index (MRCI) was calculated using the questionnaire validated for Brazilian Portuguese. This tool comprises scores for drug presentation form, dosage and additional administration directions ( *e.g* ., to dissolve or crush tablets). The final score is given as an interval scale created from summed scores, with no set threshold or maximum value.^(^^28^^)^

Adherence to diabetes treatment was assessed by direct verification of correct medication use based on prescription and participant self-report. In the case of individuals requiring assistance with medications, adherence was confirmed by caregivers.

#### Depression

Depression was evaluated in this study due to potential associations between mood disorders and poor glycemic control.^(^^29^^)^ A short version of the Geriatric Depression Scale (GDS) comprising 15 items was used. Participants scoring higher than 5 were defined as depressed.^(^^30^^)^

### Statistical analysis

Descriptive analysis was performed using measures of frequency or central tendency (categorical and interval variables, respectively). Data were expressed as means and standard deviations. Normality assessment was based on a histogram selected for parametric tests.

The primary endpoint (HbA1c value) was analyzed as a continuous variable. Associations with interval and categorical variables were determined using Pearson correlation analysis and the Student's *t* test, respectively.

Simple and forced-entry hierarchical multivariate linear regression models were then created using HbA1c value as the dependent variable. In multiple hierarchical regression models, covariates were entered in three sequential blocks according to increments in R^2^ values. The sociodemographic variables (age, sex, race, labor) were entered first. Clinical variables (MOS, disease duration, knowledge on diabetes, medication regimen complexity index, adherence to diabetes treatment, symptoms of depression, and BMI) were then entered in the second model. Finally, SAHLPA scores were entered. Missing data were accounted for in the analysis.

Next, interaction analysis was conducted to assess potential impacts of social support on the relation between health literacy and glycemic control. To determine interaction effects in regression models, MOS scores were analyzed as a continuous variable. To demonstrate relations between health literacy and HbA1c values in each social group, MOS scores were also evaluated by terciles. Short Assessment of Health Literacy for Portuguese-speaking Adults score, MOS score and the interaction variable were included in the final interaction analysis model.

Study power estimation was based on the ability of the multivariate linear regression model to predict HbA1c values. Assuming an effect size of 0.17, calculated from the R^2^ value obtained in the model with 15 variables, and considering a level of significance of 5% determined using a two-tailed test (alpha=0.05), a sample size of 166 individuals was estimated to yield a power of 93%. Power and data analysis were carried out using G Power 3.0.10 for Windows and Statistical Package for Social Science version 20.0 for Windows, respectively.

## RESULTS

A total of 398 elderly patients with DM2 were evaluated during the experimental period. Of these, 232 were not eligible to participate. Exclusion was due to HbA1c <6.5% in 20 cases. The final sample comprised 166 participants. Mean SAHLPA score was 13.3 (5.0). Inadequate health literacy was detected in 46.4% of subjects. *Short Assessment of Health Literacy for Portuguese-speaking Adults* scores did not differ significantly between individuals excluded due to HbA1c <6.5% and remaining participants (12.1 and 13.3, respectively; p=0.279). Excluded individuals used less insulin (30.0% *versus* 72.3%; p<0.001) and similar proportions of oral hypoglycemic agents (95% *versus* 91%; p=0.543). With regards to social support, the mean MOS score was 81.5 (17.9). Given MOS was treated as an interval variable, scores were divided into terciles for further analysis (0 to 84, 85 to 94 and 95; first, second and third tercile, respectively).

Clinical and sociodemographic characteristics are given in table 2 .

**Table 2:** Sociodemographic and clinical characteristics of diabetic patients

Characteristics	Total
Age, years	68.0±5.9
Female sex	104.0 (62.7)
White race	63.0 (38.0)
Manual labor	86.0 (51.8)
BCEC	20.6±6.5
Schooling, years	6.5±5.1
Married or in stable relationship	83.0 (50.0)
Social support according to MOS score	81.5±17.9
Disease duration, years	18.5±8.8
Need for help with medication	35.0 (21.1)
Insulin use	120.0 (72.3)
Use of insulin pen	9.0 (5.4)
Diabetes knowledge according to SKILLD	6.6±1.8
Adherence to diabetes medication	110.0 (66.3)
MRCI	45.7±16.1
Symptoms of depression according to GDS 15 >5	43.0 (25.9)
BMI, kg/m^2^ (6 MD)	30.3±5.4
SAHLPA score	13.3±5.0
HbA1c	8.5±1.4

Results expressed as mean ± standard deviation or n (%).

BCEC: Brazilian Criteria of Economic Classification; MOS: Medical Outcomes Study; SKILLD: Spoken Knowledge in Low Literacy Patients with Diabetes; MRCI: Medication Regimen Complexity Index; GDS: Geriatric Depression Scale; BMI: body mass index; MD: missing data; SAHLPA: Short Assessment of Health Literacy for Portuguese-speaking Adults; HbA1c: glycated hemoglobin.

Results of bivariate analysis and correlations of sociodemographic characteristics and clinical variables with HbA1c values are shown in table 3 .

**Table 3:** Associations and correlations between sociodemographic characteristics, clinical variables, Short Assessment of Health Literacy for Portuguese-speaking Adults score and glycated hemoglobin values

Characteristics		Mean HbA1c (SD)	Pearson's correlation coefficient	p value
Age			-0.148	0.057 *
Sex				0.939 †
	Female	8.5 (1.5)		
	Male	8.5 (1.2)		
Race				0.876 †
	White	8.5 (1.8)		
	Others	8.5 (1.3)		
Labor				0.076 †
	Manual	8.7 (1.5)		
	Not manual	8.3 (1.2)		
BCEC (1 MD)			-0,.136	0.080 *
Schooling, years			-0.058	0.455 *
Marital status				0.733 †
	Married or in stable relationship	8.5 (1.2)		
	Others	8.4 (1.6)		
Social support according to MOS			-0.063	0.080 *
Disease duration, years			0.081	0.455 *
Need for help with medication				0.342 †
	No	8.4 (1.3)		
	Yes	8.7 (1.5)		
Insulin use				
	No	7.8 (0.9)		<0.001 †
	Yes	8.7 (1.5)		
Diabetes knowledge according to SKILLD			-0.090	0.248 *
Adherence to diabetes medication				0.746 †
	No	8.5 (1.4)		
	Yes	8.5 (1.4)		
MRCI			0.317	<0.001 *
Symptoms of depression according to GDS 15 >5				0.016 †
	Not depressed	8.3 (1.3)		
	Depressed	8.9 (1.4)		
BMI, kg/m^2^ (6 MD)			0.192	0.015 *
SAHLPA			-0.121	0.120 *

* Pearson's correlation test;

† Student´s *t* test to compare means between two groups.

HbA1c: glycated hemoglobin; SD: standard deviation; BCEC: Brazilian Criteria of Economic Classification; MD: missing data; MOS: Medical Outcomes Study; SKILLD: Spoken Knowledge in Low Literacy Patients with Diabetes; MRCI: Medication Regimen Complexity Index; GDS: *Geriatric Depression Scale* ; BMI: body mass index; SAHLPA: Short Assessment of Health Literacy for Portuguese-speaking Adults.

The multiple linear regression model employed to test associations between glycemic control, sociodemographic characteristics, clinical variables and SAHLPA scores is described in table 4 . Age (B=-0.50; p=0.018), MRCI (B=0.024; p=0.001) and SAHLPA (B=-0.052; p=0.044) were independently associated with HbA1c values. Insulin use, use of insulin pen and marital status caused heteroscedasticity and were therefore excluded from the model. Also, MRCI provides more comprehensive data regarding insulin use. Brazilian Criteria of Economic Classification and education were also excluded from the model. Finally, only very few participants used insulin pens (n=9).

**Table 4:** Associations between glycated hemoglobin values, sociodemographic characteristics, clinical variables and Short Assessment of Health Literacy for Portuguese-speaking Adults scores detected using simple and forced-entry hierarchical multivariate linear regression models

Characteristics	No adjustment *		Model 1 † R^2^=0.029		Model 2 † R^2^=0.122		Model 3 † R^2^=0.140	
	Beta	p value	beta	p value	beta	p value	beta	p value
Age, years	-0.034	0.057	-0.043	0.022	-0.040	0.053	-0.500	0.018
Female *versus* male sex	0.017	0.939	-0.025	0.913	-0.183	0.441	-0.189	0.421
White *versus* other races	0.035	0.876	0.237	0.303	0.174	0.440	0.294	0.204
Manual *versus* not manual labor	0.381	0.076	0.492	0.028	0.231	0.335	0.120	0.621
Social support according to MOS score	-0.005	0.418			0.006	0.394	-0.009	0.197
Disease duration, years	0.013	0.302			0.017	0.226	0.018	0.204
Diabetes knowledge according to SKILLD	-0.070	0.248			0.129	0.061	-0.111	0.108
MRCI	0.027	<0.001			0.022	0.002	0.024	0.001
Adherence to diabetes medication	-0.74	0.746			0.009	0.967	0.004	0.986
Symptoms of depression according to GDS 15 >5 depressed *versus* not depressed	0.585	0.016			0.173	0.546	0.029	0.922
BMI, kg/m^2^‡	0.049	0.015			0.030	0.146	0.024	0.241
SAHLPA	-0.034	0.120					-0.052	0,044

* Univariate regression;

† hierarchical multiple linear regression using HbA1c value as the dependent variable;

‡ 6 missing data.

Model 1: p=0.067; model 2: p=0.001; model 3: p=0.001. Model 1: adjusted for sociodemographic characteristics (age, sex, race and labor); model 2: adjusted for sociodemographic characteristics and clinical variables (MOS, disease duration, diabetes knowledge, medication regimen complexity index, adherence to diabetes medication, symptoms of depression and BMI); model 3: adjusted for sociodemographic characteristics, clinical variables and SAHLPA.

MOS: *Medical Outcomes Study;* SKILLD: *Spoken Knowledge in Low Literacy Patients with Diabetes* ; MRCI: Medication Regimen Complexity Index; GDS: *Geriatric Depression Scale* ; BMI: body mass index; SAHLPA: *Short Assessment of Health Literacy for Portuguese-speaking Adults.*

**Figure 1:**
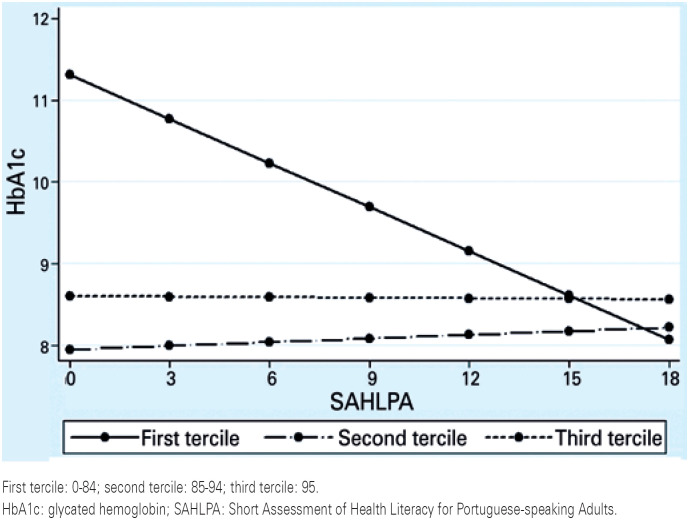
Analysis of interactions between Short Assessment of Health Literacy for Portuguese-speaking Adults score and glycated hemoglobin value according to medical outcomes study score terciles

Analysis of interactions between health literacy and social support revealed that SAHLPA score effects on glycemic control varied according to MOS (p=0.002). Figure 1 shows the linear relationship between SAHLPA score and HbA1c values.

## DISCUSSION

In this sample of elderly diabetic (DM2) patients with low levels of education and mean HbA1c of 8.5% (±1.4), functional health literacy was associated with glycemic control. Also, interaction between health literacy and social support acted as a determinant of glycemic control. Other variables related to glycemic control were age and MRCI.

Associations between low levels of health literacy and greater social support have been demonstrated in individuals with DM2. Therefore, social support may act as a neutralizing factor in the relation between health literacy and glycemic control,^(^^15^^)^ as indicated by the stronger association between SAHLPA score and glycemic control in individuals with low social support in this sample.

Findings of a meta-analysis of 28 studies, including 5,242 individuals and conducted in 2017, suggested social support is moderately associated with self-care in diabetes (r=28; 95%CI: 21-34).^(^^31^^)^ Year upon year, the ADA publications have emphasized the significance of a centralized communication-based approach accounting for patients’ beliefs and preferences, and accessing literacy, numeracy, social support and potential barriers to care whenever possible.^(^^20^^)^

Data on the complexity of pharmacological treatment are scarce in literature. Yet, this is thought to be an important factor in DM2 control, since these individuals tend to need more medications over time.^(^^16^^)^ Melchiors et al., evaluated 95 diabetic patients with a mean age of 58.5±11.2 years, and failed to find correlations between MRCI and HbA1c levels (r=0.06; p=0.56). However, that study aimed to validate the MRCI for the Portuguese language; therefore, sample size was not calculated to demonstrate associations between complexity of pharmacotherapy and glycemic control.^(^^28^^)^

Conversely, in a study conducted by Martinez et al., evaluating 235 DM2 patients with a mean age of 61.4±9.9 years, higher MRCI scores were correlated with poorer glycemic control (r=0.16; p<0.01). Despite these findings, the authors did not assess the impact of other factors, such as confounding variables ( *e.g* ., adherence).^(^^32^^)^

This study revealed an inverse relation between age and HbA1c value, suggesting a protective effect in individuals over the age of 60 years. Pharmacokinetic and pharmacodynamic changes in elderly patients may interfere with the effects of several medications, including oral antidiabetic agents and insulin. Although DM2 tends to progress to pancreatic failure over time, medication doses often should be reduced in older individuals due to the risk of hypoglycemia.^(^^16^^)^

This article makes an important contribution, since most relevant publications addressing health literacy and outcomes in diabetes were carried out in developed countries, with highly educated populations.^(^^10^^,^^33^^)^ The few studies conducted with samples comprising individuals with low socioeconomic status and low levels of schooling reported similar findings.^(^^12^^–^^14^^)^ Among previous studies, few evaluated participants with similar severity profile (mean HbA1c higher than 8.0%).^(^^8^^,^^10^^,^^11^^,^^14^^)^ Given treatment of patients with longer duration of diabetes and more severe disease is more complex, health literacy may play a more relevant role in this group. Individuals with this profile should therefore be investigated.^(^^33^^)^ The fact that this was the first study conducted with elderly DM2 patients living in a developing country, and that important sociodemographic and clinical variables, potential confounders and effect modifiers, such as social support, were included in the analysis must be emphasized. Findings of this study suggest this population profile is worthy of further investigation in future research.

This study has some limitations. Firstly, cross-sectional design does not allow establishment of causality. Secondly, although exclusion of a large number of individuals minimized potential biases regarding HbA1c levels, this may lead to superselection and loss of external validity. Selection of individuals with HbA1c higher than or equal to 6.5% was due to the u-shaped DM2 curve, where extremes represent higher mortality, given our aim was to evaluate linear relations between HbA1c and outcomes.^(^^16^^)^ To access the risk of selection bias, analysis of excluded patients (20) was carried out. Health literacy levels did not differ between groups, although there was higher proportion of insulin use in the group with higher HbA1c values. Thirdly, the most recent HbA1c value measured within the last 6 months was used as reference for good glycemic control. However, HbA1c values are somewhat variable. The fourth limitation was the inability to demonstrate which individuals participated in diabetes education groups. Nonetheless, the study was conducted at a referral center and all participants were given detailed instructions on disease management during medical visits. Also, diabetes knowledge assessment in this study was based on SKILLD. Fifthtly, adherence to lifestyle habits, a relevant aspect in the treatment of DM2, could not be examined. Still, inclusion of the covariate BMI in all models may have reflected adherence to lifestyle habits. Sixthly, the Crockoft-Gault formula was used to exclude individuals with kidney dysfunction. Despite evidence of superior performance of the Chronic Kidney Disease Epidemiology Collaboration (CKD-EPI) formula, exclusion of these individuals aimed exclusively to eliminate factors that might interfere with HbA1c measurement, and clinical outcomes associated with glomerular filtration rate were not analyzed.^(^^23^^)^ Finally, the fact that the instrument selected for health literacy assessment (SAHLPA) does not measure important domains of health literacy, such as numerical skills, must be emphasized.^(^^4^^,^^5^^)^ Other than that, SAHLPA is a user-friendly tool and is thought to be more appropriate for populations living in developing countries, because it tests more rudimentary health literacy skills.

## CONCLUSION

Health literacy, age and Medication Regimen Complexity Index score were associated with glycemic control in this study. Social support seems to modify the relation between health literacy and glycemic control. Future research with individuals with low levels of education should be conducted to explore health literacy domains other than the functional domain.
