# Supplementary material for: Association between health literacy and glycemic control in elderly patients with type 2 diabetes and modifying effect of social support
Source: Einstein (Sao Paulo). 2020 Nov 25;18:eAO5572. doi: 10.31744/einstein_journal/2020AO5572 (PMC7690930; doi:10.31744/einstein_journal/2020AO5572)
Supplement: Translation

# Translation: Associação entre alfabetismo em saúde e controle glicêmico em idosos com diabetes tipo 2 e efeito modificador do suporte social

**Authors:** Jonas Gordilho Souza, José Marcelo Farfel, Omar Jaluul, Márcia Silva Queiroz, Márcia Nery

**Affiliations:** 1 SalvadorBA Brasil Faculdade de Medicina da Bahia, Universidade Federal da Bahia, Salvador, BA, Brasil.; 2 São PauloSP Brasil Faculdade de Medicina, Universidade de São Paulo, São Paulo, SP, Brasil.; 3 São PauloSP Brasil Hospital das Clínicas, Faculdade de Medicina, Universidade de São Paulo, São Paulo, SP, Brasil.

**Keywords:** Alfabetismo em saúde, *Diabetes mellitus* tipo 2, Glicemia, Idoso, Saúde do idoso

## Abstract

**Objetivo:**

Verificar a associação entre alfabetismo em saúde inadequado, segundo o *Short Assessment of Health Literacy for Portuguese-speaking Adults* , e controle glicêmico, em pacientes idosos com diabetes tipo 2, bem como avaliar tal associação no contexto de baixo suporte social, segundo o *Medical Outcomes Study* .

**Métodos:**

Estudo transversal conduzido no centro de referência de diabetes de um hospital universitário. Os participantes foram recrutados entre pacientes com diabetes tipo 2 com idade de 60 anos ou mais, entre maio de 2013 e novembro de 2014. O desfecho primário foi o valor mais recente de hemoglobina glicada obtido nos últimos 6 meses.

**Resultados:**

Foram avaliados 398 pacientes idosos com diabetes tipo 2. Destes, 232 não foram considerados elegíveis para participar da pesquisa. A amostra final incluiu 166 participantes. Foi realizada análise de regressão linear múltipla hierárquica com as seguintes variáveis inseridas em três blocos: características sociodemográficas, variáveis clínicas e escore de alfabetismo em saúde. Realizou-se também uma análise de regressão adicional da interação entre alfabetismo em saúde e apoio social como determinante do controle glicêmico. A média de idade dos indivíduos foi 68,0 anos (desvio-padrão de 5,9). O valor médio de hemoglobina glicada foi de 8,5% (desvio-padrão de 1,4). O *Short Assessment of Health Literacy for Portuguese-speaking Adults* mostrou-se independentemente associado à hemoglobina glicada (B=-0,059; p=0,043). A interação entre suporte social e escore de alfabetismo em saúde (p=0,003) foi determinante para o controle glicêmico.

**Conclusão:**

O alfabetismo em saúde está associado ao controle glicêmico. O suporte social pode modificar a relação entre o escore de alfabetismo em saúde e o controle glicêmico.

## INTRODUÇÃO

O alfabetismo em saúde é definido como o grau individual de capacidade de obter, processar e compreender informações básicas e serviços de saúde necessários para tomar decisões adequadas relacionadas à saúde. O conceito atual inclui habilidades de comunicação e críticas, como a compreensão da dosagem de medicamentos, dos rótulos e dos exames, a busca de informações sobre saúde, a análise de riscos e benefícios, a comunicação de necessidades e a discussão de preferências.^(^^1^^)^

Baixas taxas de alfabetismo em saúde foram observadas em alguns grupos, particularmente naqueles formados por indivíduos de classe socioeconômica baixa e idosos. O alfabetismo em saúde também pode ter impacto importante em idosos com multimorbidades, que requerem terapias complexas e são mais propensos a utilizar serviços de saúde.^(^^2^^)^

Nas últimas décadas, diversos instrumentos destinados à avaliação do alfabetismo em saúde foram desenvolvidos, como o *Short Assessment of Health Literacy for Portuguese-speaking* Adults (SAHLPA). Esse teste avalia o alfabetismo funcional em saúde e é considerado mais adequado para aplicação em países em desenvolvimento, uma vez que aborda habilidades mais rudimentares relacionadas ao alfabetismo em saúde, o que facilita seu uso em indivíduos com baixo grau de escolaridade.^(^^3^^,^^4^^)^

Foram demonstradas associações entre alfabetismo em saúde inadequado e condições como capacidade limitada de compreender e seguir instruções médicas,^(^^5^^)^maior risco de hospitalização e^(^^6^^)^taxas mais altas de mortalidade.^(^^7^^)^

Devido a esses achados, a investigação de associações entre alfabetismo em saúde e controle de doenças crônicas constitui importante campo de pesquisa.^(^^8^^)^O *diabetes mellitus* tipo 2 (DM2) é um bom modelo para estudo das relações citadas, uma vez que se trata de uma doença complexa, cujo controle requer autocuidado e compreensão adequada de tratamentos farmacológicos e não farmacológicos, principalmente entre os que sofrem da doença por um longo tempo. Além disso, barreiras ao controle glicêmico, principalmente a polifarmácia e as alterações farmacodinâmicas e farmacocinéticas, podem tornar o controle do DM2 difícil em idosos.^(^^9^^)^

As possíveis relações entre alfabetismo em saúde e controle glicêmico foram investigadas por diversos pesquisadores, com resultados variáveis.^(^^10^^)^Metanálise recente revelou associações entre alfabetismo em saúde e controle glicêmico em subgrupos com DM2 e maior proporção de indivíduos com baixo grau de escolaridade.^(^^11^^)^A baixa condição socioeconômica é destacada na maioria das publicações relevantes que relatam tal associação.^(^^12^^–^^14^^)^

O baixo alfabetismo em saúde foi associado a valores de hemoglobina glicada (HbA1c) ≥8% em pacientes diabéticos idosos (razão de chance – RC=4,78; intervalo de confiança de 95% – IC95%: 1,38-16,6). Ainda assim, o controle glicêmico não foi pior entre participantes analfabetos, sugerindo que as dificuldades enfrentadas por esses indivíduos são mais facilmente identificadas por familiares e profissionais da saúde, o que pode se traduzir em maior apoio social e medidas compensatórias. Entretanto, o efeito do suporte social não foi demonstrado no estudo em questão.^(^^12^^)^Osborn et al., investigaram os efeitos de possíveis fatores determinantes do controle glicêmico em idosos com alto grau de escolaridade e concluíram que o alfabetismo em saúde tem apenas efeito indireto por meio do suporte social. Esse achado reforça a hipótese de que a associação entre alfabetismo em saúde e controle glicêmico pode ser compensada pelo apoio social.^(^^15^^)^

Poucos estudos até hoje investigaram os mecanismos por meio dos quais o apoio social pode mascarar ou modificar o efeito do alfabetismo em saúde no controle do diabetes, principalmente em pacientes idosos que vivem em países em desenvolvimento, onde o grau de escolaridade é baixo. Dada a alta complexidade do tratamento da DM2 e a maior suscetibilidade de idosos com menor grau de escolaridade ao baixo alfabetismo em saúde, este estudo foi desenvolvido para avaliar as possíveis associações entre alfabetismo funcional em saúde e controle glicêmico em pacientes idosos com DM2 e baixo grau de escolaridade residentes em um país em desenvolvimento.

## OBJETIVO

Avaliar a associação entre alfabetismo funcional em saúde ( *Short Assessment of Health Literacy for Portuguese-speaking Adults* ) e controle glicêmico em idosos diabéticos com baixa escolaridade e residentes em um país em desenvolvimento. Como objetivo secundário, propomos uma avaliação das relações entre apoio social, alfabetismo funcional em saúde e controle glicêmico em idosos diabéticos.

## MÉTODOS

### Desenho experimental, local e aspectos éticos

Estudo observacional transversal conduzido em um ambulatório público de saúde. Este estudo foi aprovado pela Comissão de Ética para Análise de Projetos de Pesquisa (CAPPesq) do Hospital das Clínicas da Faculdade de Medicina da Universidade de São Paulo (USP), sob protocolo 10639, CAAE: 15560213.9.0000.0068. Todos os pacientes assinaram um Termo de Consentimento Informado antes da inclusão.

As consultas médicas foram realizadas por médicos residentes e supervisionadas por especialistas em tratamento do diabetes. Todos os participantes tiveram igual acesso aos medicamentos antidiabéticos, fornecidos gratuitamente pelo sistema público de saúde local.

### Participantes e coleta de dados

Os participantes foram recrutados por amostragem por conveniência entre os pacientes atendidos no ambulatório de um grande centro de referência de diabetes de um hospital universitário terciário (Hospital das Clínicas da Faculdade de Medicina da USP), na cidade de São Paulo (SP). Pacientes diabéticos atendidos entre maio de 2013 e novembro de 2014 foram convidados a participar no ato das consultas médicas.

O recrutamento e a coleta de dados ficaram a cargo de um dos pesquisadores. Os pacientes foram abordados na sala de espera, antes da consulta médica, quando foram devidamente informados a respeito do estudo. Uma vez obtido o Consentimento Informado, os participantes foram entrevistados na sala de atendimento. A confidencialidade foi assegurada.

### Critérios de inclusão e exclusão

Foram adotados os seguintes critérios de inclusão: idade igual ou superior a 60 anos, fluência em português, diagnóstico de DM2 de acordo com os critérios da *American Diabetes Association* (ADA)^(^^16^^)^e dosagem recente de HbA1c (não mais de 6 meses antes da inclusão).

Foram excluídos os pacientes cujos prontuários médicos/exames revelaram:

– Menos de três visitas ao ambulatório de diabetes.
– Dosagem de HbA1c <6,5%, dado o formato em U da curva de controle glicêmico em idosos, na qual os extremos são associados à mortalidade mais elevada.^(^^16^^)^Esse critério foi estabelecido a fim de se manter uma relação linear entre valores de HbA1c e desfechos negativos.
– Deficiência de visão, audição ou fala de gravidade suficiente para afetar a resposta aos questionários. A acuidade visual foi mensurada por meio do teste de Snellen.^(^^17^^)^O valor de corte adotado para alteração visual e exclusão do estudo foi de 0,5. A capacidade auditiva foi avaliada pelo teste do sussurro.^(^^18^^)^A perda auditiva foi definida como percepção de palavras e números abaixo de 50%.
– Uso de medicações associadas ao mau controle glicêmico; histórico de cirurgia bariátrica ou participação em ensaios clínicos relacionados ao tratamento do diabetes; e síndrome da fragilidade, visto que o alvo de controle glicêmico proposto para idosos frágeis é menos estrito do que o proposto para indivíduos não frágeis.^(^^16^^)^A síndrome da fragilidade foi definida de acordo com os seguintes critérios: perda não intencional de peso de mais de 5% no último ano; incapacidade de sentar e levantar de uma cadeira cinco vezes sem auxílio; perda de energia, definida pela pergunta: “Você se sente cheio de energia?”. Indivíduos que satisfizeram dois dos três critérios acima foram definidos como frágeis.^(^^19^^)^
– Perda cognitiva definida como défice apontado pelo Miniexame do Estado Mental (MEEM). Os escores do MEEM foram combinados com o grau de escolaridade. Os seguintes valores de corte foram aplicados de acordo com o tempo de educação formal:^(^^20^^)^analfabeto e escore MEEM igual ou inferior a 21; 1 a 5 anos de estudo e escore MEEM igual ou inferior a 22; 6 a 11 anos de estudo e escore MEEM igual ou inferior a 23; alto grau de escolaridade (12 anos ou mais) e escore MEEM igual ou inferior a 24.
– Diagnóstico prévio de confusão mental, distúrbios psicóticos, mania, alcoolismo ou uso de drogas; anomalias laboratoriais que possam interferir no método de dosagem de HbA1c, como disfunção tireoidiana (hormônio tireoestimulante <0,1 ou >10mU/L),^(^^21^^)^anemia (hemoglobina <11mg/dL e <10mg/dL em homens e mulheres, respectivamente),^(^^22^^)^doença renal crônica com taxa estimada de filtração glomerular abaixo de 30mL/min/1,73m^2^, definida pela fórmula de Cockcroft-Gault^(^^23^^)^e doença hepática parenquimatosa classificada como Child-Pugh B ou C.^(^^24^^)^

### Ferramentas de coleta de dados e variáveis

#### Características demográficas e dados de exame físico e clínico

Os primeiros dados extraídos dos prontuários médicos ou entrevistas foram o tempo decorrido desde o diagnóstico do diabetes e as drogas prescritas em uso. Foram extraídos também os seguintes dados demográficos: idade, sexo, estado civil (casado ou não), anos de estudo, raça conforme determinada pelo entrevistador (branco ou não branco) e ocupação prévia (trabalho manual ou não). A condição socioeconômica foi determinada com base no Critério de Classificação Econômica Brasil (CCEB) e expressa na forma de intervalos.^(^^25^^)^

As dosagens de HbA1c dos últimos 6 meses foram extraídas dos prontuários médicos. No nosso serviço, a dosagem de HbA1c é feita pelo método de cromatografia líquida de alto desempenho (CLAE) certificado pelo *National Glycohemoglobin Standardization Program* .

A avaliação antropométrica foi realizada empregando-se uma balança digital (Lucastec, Brasil). O índice de massa corporal (IMC) foi calculado a partir das medidas de altura e peso corporal.

Os instrumentos utilizados neste estudo encontram-se descritos na tabela 1 .

**Tabela 1:** Instrumentos usados para avaliação e interpretação das variáveis

Instrumento	Versão em português	Intervalo	Interpretação
SAHLPA *short version* – avalia o alfabetismo em saúde	Apolinario et al.^(^^4^^)^	0-18	Analisado na forma de variável intervalar. Pode ser analisado como variável dicotômica; escores <14 definem baixo alfabetismo em saúde
SKILLD – avalia o conhecimento sobre o diabetes	Souza et al.^(^^26^^)^	0-10	Analisado na forma de variável intervalar. Não há valor de corte
MOS – avalia 5 dimensões do apoio social	Griep et al.^(^^27^^)^	0-95	Analisado na forma de variável intervalar; dados divididos em tercis. Não há valor de corte
ICFT – avalia a complexidade do tratamento farmacológico	Melchiors et al.^(^^28^^)^	Até 0	Analisado na forma de variável intervalar. Não há valor de corte
GDS-15 – avalia sintomas de depressão	Almeida et al.^(^^30^^)^	0-15	Participantes com escores >5 foram considerados deprimidos

SAHLPA: *Short Assessment of Health Literacy for Portuguese-speaking adults - short version.* SKILLD: *Spoken Knowledge in Low Literacy Patients with Diabetes* ; MOS: *Medical Outcomes Study* ; ICFT: Índice de Complexidade da Farmacoterapia; GDS-15: *Geriatric Depression Scale* .

#### Alfabetismo em saúde e conhecimento sobre o diabetes

O alfabetismo em saúde foi avaliado com base no SAHLPA. Essa ferramenta avalia o alfabetismo funcional em saúde, por meio da leitura de termos médicos que devem ser correlacionados com duas outras palavras. Ao ler a palavra “osteoporose”, por exemplo, o entrevistado deve optar entre as alternativas “osso” e “músculo”.^(^^3^^,^^4^^)^O SAHLPA foi selecionado por ser um instrumento adequadamente validado para o português e um teste simples, o que facilita a aplicação em indivíduos com alfabetização rudimentar. A versão curta do teste se destina a idosos, uma vez que tem a mesma acurácia da versão longa (50 itens) e maior facilidade de aplicação no contexto da avaliação geriátrica ampla. Neste estudo, os escores do SAHLPA foram analisados na forma de intervalos.

O conhecimento sobre o diabetes foi mensurado com base no teste SKILLD. Essa ferramenta consiste de dez perguntas relacionadas à compreensão da doença. Uma vez que se trata de uma ferramenta de administração oral, a capacidade de leitura tem pouca importância, daí a aplicabilidade em populações com baixos graus de escolaridade. Os escores SKILLD variam de zero a 100% e quanto mais alto o escore, melhor a compreensão do indivíduo a respeito das questões relacionadas ao diabetes.^(^^26^^)^

#### Suporte social

A necessidade de ajuda com as medicações foi investigada nos prontuários médicos. Os participantes foram caracterizados como necessitados de ajuda para organizar, lembrar ou gerenciar a tomada das medicações ou como totalmente dependentes.

A ferramenta *Medical Outcomes Study* (MOS) também foi utilizada. Esse instrumento avalia o apoio social recebido pelo paciente em cinco dimensões, com escores variando de 1 (nunca) a 5 (sempre).^(^^27^^)^O escore final vai de zero a 95, sendo 95 correspondente ao melhor apoio social possível. Não há ponto de corte relatado na literatura. Desse modo, os escores foram divididos em tercis para a análise de interações.

### Uso de medicações e adesão

O Índice de Complexidade da Farmacoterapia (ICFT) foi calculado empregando-se a versão brasileira validada do questionário. Essa ferramenta inclui escores para forma de apresentação da droga, dosagem e informações adicionais, como instruções para dissolver ou esmagar comprimidos. O escore final é expresso por meio de um intervalo obtido a partir da soma dos demais escores, sem limiar ou valor máximo determinado.^(^^28^^)^

A adesão ao tratamento do diabetes foi avaliada por conferência direta do uso correto das medicações com base na prescrição e no autorrelato. No caso de indivíduos com necessidade de ajuda com as medicações, o dado foi confirmado junto aos cuidadores.

#### Depressão

Dada a possível associação entre transtornos de humor e mau controle glicêmico,^(^^29^^)^a depressão foi avaliada neste estudo. Uma versão curta da *Geriatric Depression Scale* (GDS) contendo 15 itens foi utilizada. Os participantes com escores acima de 5 foram considerados deprimidos.^(^^30^^)^

### Análise estatística

Inicialmente, foi realizada a análise descritiva empregando-se medidas de frequência e tendência central (variáveis categóricas e intervalares, respectivamente). Os dados foram expressos na forma de médias e desvios-padrão. A avaliação da normalidade foi baseada em um histograma selecionado para testes paramétricos.

O desfecho primário (valor de HbA1c) foi avaliado como variável contínua. As associações com as variáveis intervalares e categóricas foram determinadas empregando-se a análise de correlação de Pearson e o teste *t* de Student, respectivamente.

Modelos de regressão linear hierárquica multivariada simples e de entrada forçada foram, então, criados adotando-se o valor de HbA1c como a variável dependente. Nos modelos de regressão múltipla hierárquica, as covariáveis foram inseridas em três blocos sequenciais, de acordo com o incremento do valor de R^2^. As variáveis sociodemográficas (idade, sexo, raça e trabalho) foram inseridas primeiro. As variáveis clínicas (MOS, duração da doença, conhecimento sobre o diabetes, índice de complexidade da farmacoterapia, a adesão ao tratamento do diabetes, sintomas de depressão e IMC) foram incluídas no segundo modelo. Finalmente, a variável SAHLPA foi acrescentada. Os dados faltantes foram considerados na análise.

A seguir, uma análise de interações foi realizada para avaliar os possíveis efeitos do suporte social sobre a relação entre alfabetismo em saúde e controle glicêmico. Para determinar os efeitos de interações nos modelos de regressão, os escores do MOS foram avaliados como variável contínua. A fim de se demonstrar a relação entre alfabetismo em saúde e valor de HbA1c em cada grupo social, o MOS também foi avaliado por tercis. As variáveis SAHLPA, MOS e de interação foram incluídas no modelo final.

A estimativa do poder do estudo foi baseada na capacidade de o modelo de regressão linear multivariada prever o valor de HbA1c. Considerando um tamanho de efeito de 0,17, calculado a partir do valor de R^2^obtido no modelo com 15 variáveis, e um nível bicaudal de significância de 5% (alfa=0,05), estimou-se que uma amostra com 166 indivíduos forneceria um poder de 93%. As análises de poder e de dados foram conduzidas empregando-se o G Power 3.0.10 para Windows e a versão 20.0 do (SPSS) para Windows, respectivamente.

## RESULTADOS

Ao todo, 398 pacientes idosos com DM2 foram avaliados durante o período experimental. Destes, 232 não foram considerados elegíveis. A exclusão foi devido a valores de HbA1c <6,5% em 20 casos. A amostra final teve 166 participantes. O SAHLPA médio foi 13,3 (5,0), e o baixo alfabetismo em saúde foi detectado em 46,4% da amostra. Não houve diferença significativa entre o SAHLPA dos indivíduos excluídos devido a valores de HbA1c <6,5% e o dos demais participantes (12,1 e 13,3, respectivamente; p=0,279). O uso de insulina foi menos comum entre indivíduos excluídos (30,0% *versus* 72,3%; p<0,001), e a proporção de uso de agentes hipoglicemiantes orais foi semelhante (95% *versus* 91%; p=0,543). Com relação ao suporte social, o MOS médio foi 81,5 (17,9). Como o MOS foi tratado como variável intervalar, optou-se pela divisão em tercis nas análises adicionais (0 a 84, 85 a 94 e 95, para primeiro, segundo e terceiro tercil, respectivamente).

As características clínicas e sociodemográficas dos pacientes diabéticos encontram-se descritas na tabela 2 .

**Tabela 2:** Características sociodemográficas e clínicas dos pacientes diabéticos

Características	Total
Idade, anos	68,0±5,9
Sexo feminino	104,0 (62,7)
Raça branca	63,0 (38,0)
Trabalho manual	86,0 (51,8)
CCEB	20,6±6,5
Escolaridade, anos	6,5±5,1
Casado ou em relação estável	83,0 (50,0)
Apoio social segundo o MOS	81,5±17,9
Duração da doença, anos	18,5±8,8
Necessidade de ajuda com a medicação	35,0 (21,1)
Uso de insulina	120,0 (72,3)
Uso de caneta de insulina	9,0 (5,4)
Conhecimento sobre o diabetes segundo o SKILLD	6,6±1,8
Adesão do tratamento do diabetes, não	110,0 (66,3)
ICFT	45,7±16,1
Sintomas de depressão segundo a GDS 15 >5	43,0 (25,9)
IMC, kg/m^2^(6 MD)	30,3±5,4
SAHLPA	13,3±5,0
HbA1c	8,5±1,4

Resultados expressos por média±desvio padrão ou n (%)

CCEB: Critério de Classificação Econômica Brasil; MOS: *Medical Outcomes Study;* SKILLD: *Spoken Knowledge in Low Literacy Patients with Diabetes* ; ICFT: Índice de Complexidade da Farmacoterapia; GDS: *Geriatric Depression Scale* ; IMC: índice de massa corporal; MD: missing data; SAHLPA: *Short Assessment of Health Literacy for Portuguese-speaking Adults* ; HbA1c: hemoglobina glicada.

A tabela 3 descreve os resultados da análise bivariada, as correlações entre as características sociodemográficas e clínicas e os valores de HbA1c.

**Tabela 3:** Associações e correlações entre características sociodemográficas, variáveis clínicas, escore obtido no *Short Assessment of Health Literacy for Portuguese-speaking* Adults e valores de hemoglobina glicada

		HbA1c média±DP	Coeficiente de correlação de Pearson	Valor de p
Idade			-0,148	0,057 *
Sexo				0,939 †
	Feminino	8,5 (1,5)		
	Masculino	8,5 (1,2)		
Raça				0,876 †
	Branca	8,5 (1,8)		
	Outras	8,5 (1,3)		
Trabalho				0,076 †
	Manual	8,7 (1,5)		
	Não manual	8,3 (1,2)		
CCEB (1 MD)			-0,136	0,080 *
Escolaridade, anos			-0,058	0,455 *
Estado civil				0,733 †
	Casado ou em relação estável	8,5 (1,2)		
	Outros	8,4 (1,6)		
Suporte social de acordo com o MOS			-0,063	0,080 *
Duração da doença, anos			0,081	0,455 *
Necessidade de ajuda com a medicação				0,342 †
	Não	8,4 (1,3)		
	Sim	8,7 (1,5)		
Uso de insulina				
	Não	7,8 (0,9)		<0,001 †
	Sim	8,7 (1,5)		
Conhecimento sobre o diabetes de acordo com o SKILLD			-0,090	0,248 *
Adesão ao tratamento do diabetes				0,746 †
	Não	8,5 (1,4)		
	Sim	8,5 (1,4)		
ICFT			0,317	<0,001 *
Sintomas de depressão de acordo com a GDS 15 >5				0,016 †
	Não deprimido	8,3 (1,3)		
	Deprimido	8,9 (1,4)		
IMC, kg/m^2^(6 MD)			0,192	0,015 *
SAHLPA			-0,121	0,120 *

* teste de correlação de Pearson;

† teste *t* de Student para comparação de médias entre dois grupos.

HbA1c: hemoglobina glicada; DP: desvio-padrão; CCEB: Critério de Classificação Econômica Brasil; MD: missing data; MOS: *Medical Outcomes Study;* SKILLD: *Spoken Knowledge in Low Literacy Patients with Diabetes* ; ICFT: Índice de Complexidade da Farmacoterapia; GDS: *Geriatric Depression Scale* ; IMC: índice de massa corporal; SAHLPA: *Short Assessment of Health Literacy for Portuguese-speaking Adults* .

O modelo de regressão linear múltipla do controle glicêmico ajustado para características sociodemográficas, variáveis clínicas e SAHLPA encontra-se descrito na tabela 4 . As variáveis idade (B=-0,50; p=0,018), ICFT (B=0,024, p=0,001) e SAHLPA (B=-0,052, p=0,044) mostraram-se independentemente associadas aos valores de HbA1c. Dentre as variáveis analisadas, o uso de insulina, o uso de caneta de insulina e o estado civil causaram heterocedasticidade e foram excluídas do modelo. Além disso, o ICFT forneceu dados mais abrangentes no que se referiu ao uso da insulina. As variáveis CCEB e escolaridade também foram excluídas do modelo. Finalmente, o número de participantes que utilizavam a caneta foi muito baixo (n=9).

**Tabela 4:** Associações entre valores de hemoglobina glicada e características sociodemográficas, variáveis clínicas e escores obtidos no *Short Assessment of Health Literacy for Portuguese-speaking Adults* detectadas por meio dos modelos de regressão linear hierárquica multivariada simples e de entrada forçada

Características	Sem ajuste *		Modelo 1 † R^2^=0,029		Modelo 2 † R^2^=0,122		Modelo 3 † R^2^=0,140	
	Beta	Valor de p	Beta	Valor de p	Beta	Valor de p	Beta	Valor de p
Idade, anos	-0,034	0,057	-0,043	0,022	-0,040	0,053	-0,500	0,018
Sexo feminino *versus* masculino	0,017	0,939	-0,025	0,913	-0,183	0,441	-0,189	0,421
Raça branca *versus* outras	0,035	0,876	0,237	0,303	0,174	0,440	0,294	0,204
Trabalho manual *versus* não manual	0,381	0,076	0,492	0,028	0,231	0,335	0,120	0,621
Apoio social segundo o MOS	-0,005	0,418			0,006	0,394	-0,009	0,197
Duração da doença, anos	0,013	0,302			0,017	0,226	0,018	0,204
Conhecimento sobre o diabetes segundo o SKILLD	-0,070	0,248			0,129	0,061	-0,111	0,108
IFCT	0,027	<0,001			0,022	0,002	0,024	0,001
Adesão ao tratamento do diabetes	-0,74	0,746			0,009	0,967	0,004	0,986
Sintomas de depressão segundo a GDS 15 >5 deprimido *versus* não deprimido	0,585	0,016			0,173	0,546	0,029	0,922
IMC, kg/m^2^‡	0,049	0,015			0,030	0,146	0,024	0,241
SAHLPA	-0,034	0,120					-0,052	0,044

* Regressão univariada;

† regressão linear múltipla hierárquica adotando o valor de HbA1c como variável dependente;

‡ 6 NA.

Modelo 1: p=0,067; modelo 2: p=0,001; modelo 3: p=0,001. Modelo 1: ajustado para características sociodemográficas (idade, sexo, raça, trabalho); modelo 2: ajustado para características sociodemográficas e variáveis clínicas (MOS, duração da doença, conhecimento do diabetes, ICFT, adesão ao tratamento do diabetes, sintomas de depressão, IMC); modelo 3: ajustado para características sociodemográficas, variáveis clínicas e SAHLPA.

MOS: *Medical Outcomes Study;* SKILLD: *Spoken Knowledge in Low Literacy Patients with Diabetes* ; ICFT: Índice de Complexidade da Farmacoterapia; GDS: *Geriatric Depression Scale* ; IMC: índice de massa corporal; SAHLPA: *Short Assessment of Health Literacy for Portuguese-speaking Adults.*

Primeiro tercil: 0-84; segundo tercil: 85-94; terceiro tercil: 95.

HbA1c: hemoglobina glicada; SAHLPA: *Short Assessment of Health Literacy for Portuguese-speaking Adults* .

A análise das interações entre alfabetismo em saúde e apoio social revelaram que o efeito do escore SAHLPA sobre o controle glicêmico foi modificado de acordo com o escore MOS (p=0,002). A figura 1 mostra a relação linear entre escore SAHLPA e HbA1c.

**Figura 1:** Análise das relações entre escores obtidos no *Short Assessment of Health Literacy for Portuguese-speaking Adults* e valores de hemoglobina glicada, de acordo com os tercis de escores obtidos no *Medical Outcomes Study*

## DISCUSSÃO

Nesta amostra de pacientes idosos com DM2, baixo grau de escolaridade e HbA1c média de 8,5% (±1,4), o alfabetismo em saúde funcional mostrou-se associado ao controle glicêmico. A interação entre alfabetismo em saúde e suporte social como determinante do controle glicêmico também foi detectada. Outras variáveis relacionadas ao controle glicêmico foram idade e ICFT.

Associações entre baixos níveis de alfabetismo em saúde e suporte social foram demonstradas em indivíduos com DM2. Assim, o apoio social pode agir como fator de neutralização na relação entre alfabetismo em saúde e controle glicêmico,^(^^15^^)^conforme indicado pela associação mais robusta entre SAHLPA e controle glicêmico em indivíduos com baixo nível de suporte social nesta amostra.

Os resultados de metanálise de 28 estudos, incluindo 5.242 indivíduos e realizada em 2017, sugerem associações moderadas entre suporte social e autocuidado em diabetes (r=28; IC95%: 21-34).^(^^31^^)^Ano após ano, a publicações da ADA enfatizam a importância de uma abordagem centralizada baseada na comunicação, que respeite as crenças e preferências dos pacientes e avalie o alfabetismo em saúde, o numeramento, o apoio social e as possíveis barreiras ao cuidado, sempre que possível.^(^^20^^)^

Dados sobre a complexidade do tratamento farmacológico são escassos na literatura, porém acredita-se que esse seja um fator importante no controle da DM2, uma vez que tais indivíduos tendem a necessitar de mais medicamentos ao longo do tempo.^(^^16^^)^Melchiors et al., avaliaram 95 pacientes diabéticos com idade média de 58,5±11,2 anos e não encontraram correlações entre ICFT e níveis de HbA1c (r=0,06; p=0,56). Entretanto, o estudo em questão foi realizado para fins de validação do ICFT para a língua portuguesa, de modo que o tamanho da amostra não foi calculado para demonstrar associações entre complexidade da farmacoterapia e controle glicêmico.^(^^28^^)^

Em contrapartida, estudo realizado por Martinez et al., com 235 pacientes com DM2 e idade média de 61,4±9,9 mostrou correlação entre ICFT mais altos e pior controle glicêmico (r=0,16; p<0,01). Apesar desses achados, os autores não avaliaram o impacto de outros fatores, como variáveis de confusão (a adesão, por exemplo).^(^^32^^)^

Este estudo revelou relação inversa entre idade e valor de HbA1c, sugerindo efeito protetor em indivíduos com mais de 60 anos de idade. Sabe-se que alterações farmacocinéticas e farmacodinâmicas podem afetar o efeito de diversos medicamentos em idosos, incluindo os agentes antidiabéticos orais e a insulina. Embora a DM2 tenda a progredir para insuficiência pancreática ao longo do tempo, a redução da dose da medicação costuma ser necessária em idosos, devido ao risco de hipoglicemia.^(^^16^^)^

Este artigo traz contribuições importantes, uma vez que a maioria das publicações relevantes que abordam o alfabetismo em saúde e os desfechos no diabetes são provenientes de países desenvolvidos, cujas populações têm alto nível de escolaridade.^(^^10^^,^^33^^)^Os poucos estudos conduzidos com amostras caracterizadas por baixo nível socioeconômico e baixo grau de escolaridade obtiveram resultados semelhantes ao desta pesquisa.^(^^12^^–^^14^^)^Dentre os estudos prévios, poucos avaliaram participantes com perfil de gravidade semelhante (HbA1c média acima de 8,0%).^(^^8^^,^^10^^,^^11^^,^^14^^)^O tratamento de pacientes com diabetes de maior duração e gravidade é mais complexo, e o alfabetismo em saúde pode ter papel mais relevante nesse grupo.^(^^33^^)^Daí a importância de se avaliarem indivíduos com esse perfil. Cabe enfatizar que este foi o primeiro estudo realizado com idosos com DM2 residentes em país em desenvolvimento e no qual variáveis sociodemográficas e clínicas importantes, potenciais fatores de confusão e modificadores de efeito, como apoio social, foram incluídos na análise. Os resultados deste estudo sugerem que esse perfil populacional é digno de maior exploração em pesquisas futuras.

Este estudo tem algumas limitações. Em primeiro lugar, o desenho experimental transversal não permite o estabelecimento de causalidade. Em segundo lugar, embora a exclusão de grande número de indivíduos tenha minimizado o viés relacionado aos valores de HbA1c, isso pode ser causa de superseleção e perda de validade externa. Indivíduos com HbA1c maior ou igual a 6,5% foram selecionados devido ao formato de U da curva de DM2, cujos extremos representam mortalidade mais alta, enquanto o objetivo era avaliar relações lineares entre HbA1c e desfechos.^(^^16^^)^Para avaliar o risco de viés de seleção, foi realizada a análise dos 20 pacientes excluídos. Os níveis de alfabetismo em saúde não diferiram entre os grupos, apesar de maior proporção de uso de insulina no grupo com os valores mais elevados de HbA1c. Em terceiro lugar, o valor mais recente de HbA1c dosado nos últimos 6 meses foi empregado como referência de bom controle glicêmico; porém, sabe-se que os valores HbA1c podem variar. A quarta limitação foi a incapacidade de demonstrar quais indivíduos participaram dos grupos de educação em diabetes. Entretanto, o estudo foi conduzido em um centro de referência, e todos os participantes receberam orientações detalhadas sobre o tratamento da doença durante as consultas. Além disso, neste estudo, a avaliação do conhecimento sobre o diabetes foi baseada no SKILLD. Em quinto lugar, não foi possível avaliar a adesão a estilos de vida – um aspecto relevante no tratamento da DM2 –, porém a covariável IMC foi considerada em todos os modelos, o que pode ter refletido a adesão a estilos de vida. Em sexto lugar, a fórmula de Crockoft-Gault foi empregada para excluir indivíduos com disfunção renal. Apesar das evidências a favor do uso da fórmula desenvolvida pela *Chronic Kidney Disease Epidemiology Collaboration* (CKD-EPI), a exclusão desses indivíduos teve por intuito apenas a eliminação de fatores que pudessem interferir na dosagem de HbA1c, e os desfechos clínicos associados à taxa de filtração glomerular não foram analisados.^(^^23^^)^Finalmente, o fato de o instrumento selecionado para avaliação do alfabetismo em saúde (SAHLPA) não mensurar domínios importantes, como habilidades numéricas, deve ser considerado.^(^^4^^,^^5^^)^Fora isso, o SAHLPA é uma ferramenta de fácil aplicação e considerada a mais adequada para uso em populações de países em desenvolvimento, pois testa funções mais rudimentares associadas ao alfabetismo em saúde.

## CONCLUSÃO

O alfabetismo em saúde, a idade e o Índice de Complexidade da Farmacoterapia mostraram-se associados ao controle glicêmico. O suporte social parece modificar a relação entre alfabetismo em saúde e controle glicêmico. Estudos futuros com indivíduos com baixo grau de escolaridade devem ser realizados, para explorar outros domínios do alfabetismo em saúde, além do funcional.
